# Case Report: A case of borderline lepromatous leprosy and literature review—key clues to expand clinical diagnostic thinking

**DOI:** 10.3389/fmed.2025.1636112

**Published:** 2025-09-16

**Authors:** Li Lu, Lei Wang, Kui Wei, Xian-wen Wei, Ying-jian Hua

**Affiliations:** ^1^Department of Dermatology, Pu'er People’s Hospital, Pu'er, China; ^2^Department of Pathology, Pu'er People's Hospital, Pu'er, China; ^3^Lancang County Center for Disease Control and Prevention, Pu'er, China; ^4^Infectious Disease Hospital, Pu'er People's Hospital, Pu'er, China

**Keywords:** borderline lepromatous leprosy, case report, edema, erythema, atypical

## Abstract

**Background:**

Leprosy, also known as Hansen’s disease, is a chronic infectious disease caused by *Mycobacterium leprae*. It primarily affects the skin, peripheral nerves, mucous membranes, and the eyes. If left untreated, it can lead to severe nerve damage, deformities, and permanent disabilities. According to a report by the World Health Organization in 2023, there were approximately 180,000 new cases globally. From 2014 to 2023, the total number of new cases worldwide decreased by 14.6%. Although certain achievements have been made in the prevention and control of leprosy, there are still deep-rooted misunderstandings and prejudices regarding leprosy in society. Consequently, patients and their families suffer from discrimination and exclusion in many aspects of life, making it difficult for them to truly integrate into society.

**Case presentation:**

A 52-year-old Dai pepole woman presented with the chief complaints of “generalized edema for 6 months and erythema and numbness of the hands and feet for 3 months.” Through histopathological examination, acid-fast staining, and Bacterial Index (BI) testing, she was diagnosed with borderline lepromatous leprosy. She was administered a multi-bacillary multidrug therapy (MDT-MB) regimen for anti-leprosy treatment.

**Conclusion:**

We report a case of borderline lepromatous leprosy with generalized swelling as the initial symptom and review and analyze misdiagnosed and rare cases reported in the literature. The aim is to provide clinicians with more diagnostic ideas and reference bases and to promote the further development of leprosy prevention and control work.

## Introduction

In 1873, the Norwegian doctor Gerhard Hansen’s disease made an important discovery in the history of medicine, and first identified *Mycobacterium leprae* as the pathogen causing leprosy. Therefore, leprosy is also known as “Hansen’s disease” (1).

Leprosy is a chronic infectious disease caused by *Mycobacterium leprae* and Mycobacterium lepromatosis that primarily affects the skin and peripheral nerves. However, it can also involve mucous membranes, eyes, and bones, leading to a wide range of clinical presentations. The exact transmission mechanisms of *M. leprae* are not fully understood; however, evidence indicates that the disease primarily spreads through the upper respiratory tract. Prolonged close contact with an untreated patient with multibacillary leprosy, likely via infectious aerosols, is considered a key factor in transmission. Additionally, *Mycobacterium leprae* prefers to multiply in cooler areas, typically around a mean surface temperature of 27.4 °C. The decrease in intraoral temperature was mainly due to the bacilli initially invading the nasal passages, causing obstruction. This leads individuals to breathe through the mouth, allowing incoming air to lower oral temperature (2).

Data from the World Health Organization (WHO) in 2023 show that there are approximately 180,000 new cases globally, with an incidence rate of 22.7 per million, an increase of 5% compared to 2022. The Southeast Asian region had the highest proportion of new cases (71.9%), whereas the European region had the lowest (<1%). From 2014 to 2023, the number of new cases globally decreased by 14.6% overall. In China, 315 new cases were reported, with an incidence rate of 0.2 per million population. According to a survey by Sun Peiwen et al., by the end of 2020, there were 1,893 registered leprosy patients in China, a significant decrease compared with 6,032 cases in 2010 (3). Although substantial achievements have been made in the field of leprosy prevention and control, there are deep-rooted misunderstandings and prejudices against leprosy in society. As a result, patients and their families may still suffer from discrimination and exclusion in many aspects of life and find it difficult to integrate into society.

In view of this, it is of great significance to better understand the various clinical features of leprosy, especially the presentation of atypical cases, to improve the accuracy of clinical diagnosis, optimize treatment, and reduce social discrimination. This article reports a case of borderline lepromatous leprosy initially presenting with generalized swelling, aiming to provide guidance to improve diagnostic clarity as a reference basis for clinicians and to promote further development of leprosy prevention and control.

## Case presentation

The patient, a 52-year-old female farmer, presented with a six-month history of generalized edema and erythema on the trunk and extremities, along with numbness in the hands and feet in the previous 3 months. Six months previously, without any obvious precipitating factors, the patient reported the development of edema of the face, trunk, and extremities. There was also mild numbness in the hands and feet, yet no pain was experienced; therefore, she did not seek medical advice at that time. Over the subsequent 3 months, erythema emerged in the lumbar region, accompanied by mild pruritus, but no stinging sensation. The number of skin lesions progressively increased and spread to the extremities, buttocks, abdomen, back, neck, and face. Moreover, numbness in the hands and feet was more pronounced. The patient visited a local rural hospital (the specific diagnostic and therapeutic measures were unclear). Following a comprehensive biochemical profile, complete blood count, and abdominal and cardiac B-mode ultrasonography, no remarkable abnormalities were observed. To pursue further diagnosis and treatment, the patient attended our department’s outpatient clinic in May 2023.

Throughout the course of the disease, the patient had good spirits. Her diet, sleep, urination, and defecation were normal with no significant weight changes. She had a history of hypertension, but irregularly took antihypertensive medication. She denied any history of infectious diseases, such as tuberculosis and hepatitis B, coronary heart disease, or diabetes. There was no family history of leprosy and the patient had no known drug allergies.

**Figure 1:**
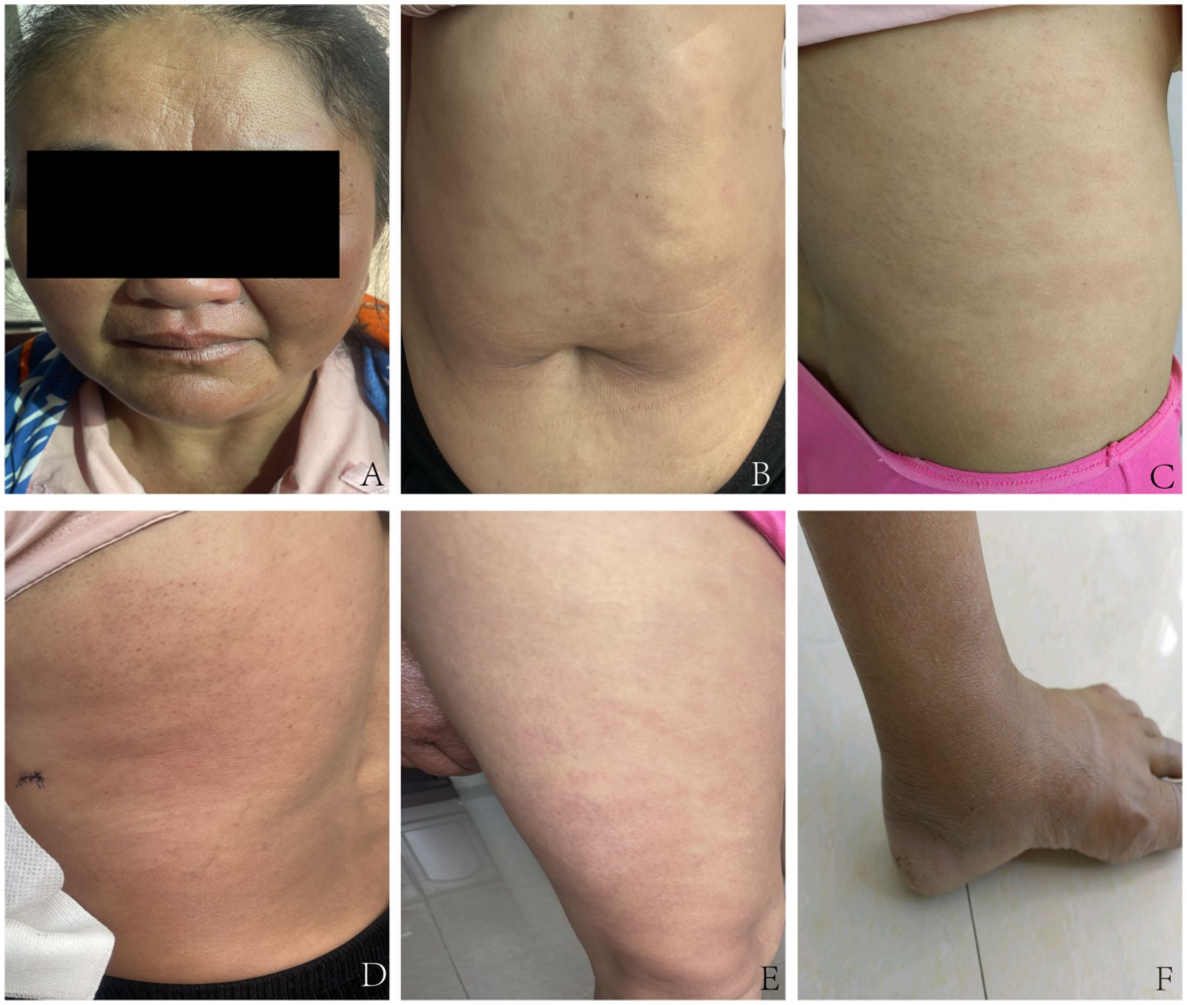
Pre-treatment clinical images of the patient. **(A)** Facial examination: Mild swelling of the face and earlobes, with infiltrative pale erythematous macules. **(B)** Trunk examination: Multiple irregular pale erythematous macules of varying sizes are visible, accompanied by mild swelling. **(C)** Lumbar and back region: Multiple pale erythematous macules are visible, with a dry surface and fine scales. **(D)** Another area of the lumbar and back region: Scattered erythematous macules adjacent to the infiltrated plaque. **(E)** Thigh examination displays several pale erythematous patches with indistinct margins. **(F)** Ankle examination: Evident pitting edema.

On specialized physical examination, the patient had scattered, variably sized, round, oval, irregular hypopigmented macules across the face, neck, trunk, and extremities. A large infiltrated plaque was noted in the lumbar area along with mild facial and earlobe swelling. The lesions had blurred borders and dry surfaces on some scales (Figures 1A–F). The skin in some lesional areas was slightly indurated, with reduced elasticity. The dorsal surfaces of the hands, lower legs, and ankles showed evident edema with mild pitting features. No thickened nerves were palpable in or around the lesions, and the warm and pain sensations were normal. The patient exhibited normal vision, complete eyelid closure, active blinking, and no conjunctival redness or swelling.

Thickening of the superficial nerves was not observed. Muscle atrophy or ataxia was not observed. The abdominal wall reflexes were normal. No deformities were observed in the extremities. The bilateral biceps brachial, triceps brachial, knee tendon, and Achilles tendon reflexes were normal. The bilateral Babinski, Gordon, and Oppenheim signs were all negative.

### Laboratory tests

**Figure 2:**
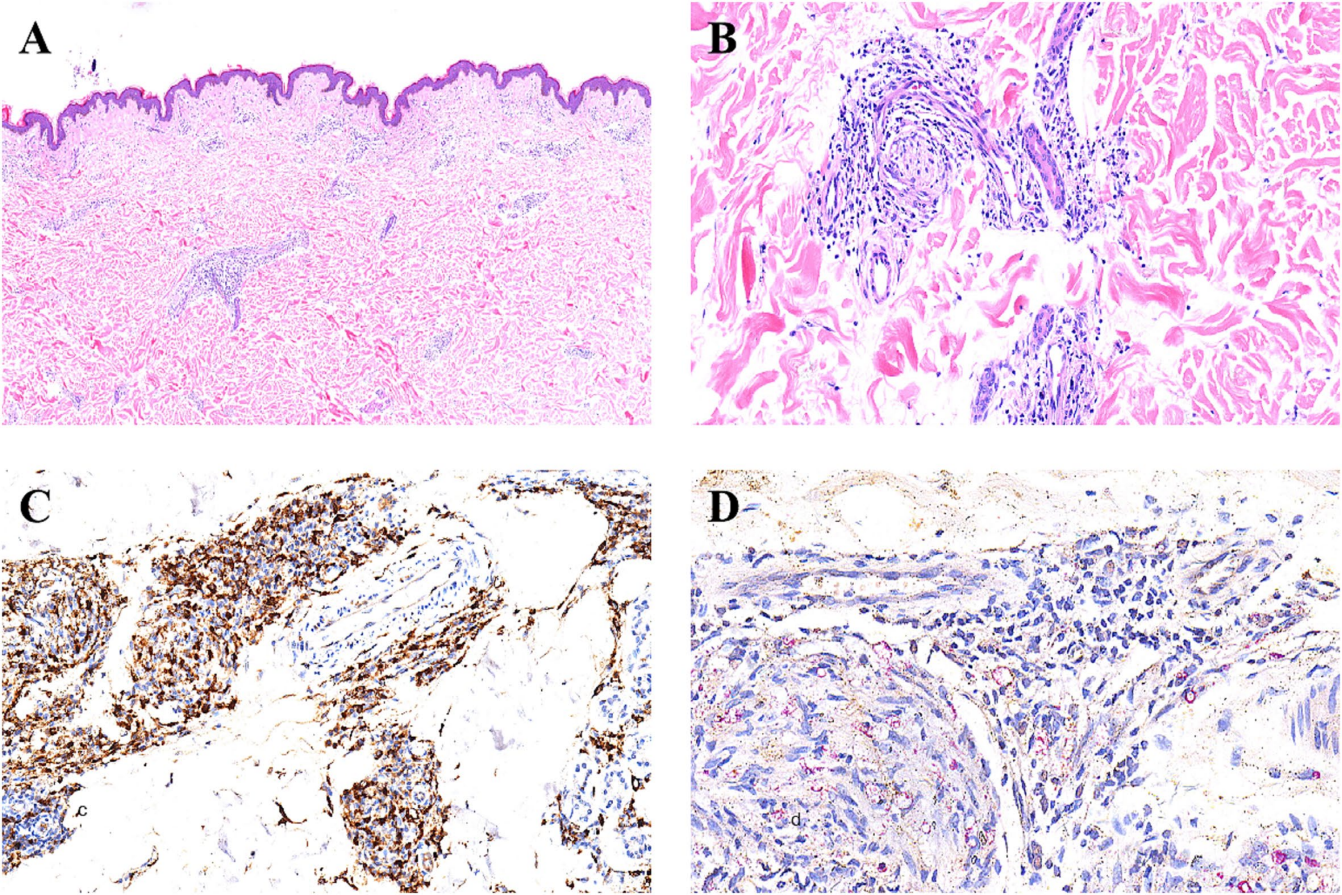
Histopathological findings. **(A)** HE staining shows focal histiocyte hyperplasia between the dermal layers of the skin lesions, and a reduction in skin appendages (hematoxylin-eosinstain, original magnification × 4). **(B)** HE staining reveals that the hyperplastic histiocytes within the dermis surround the remaining skin appendages and nerves (hematoxylin-eosinstain, original magnification × 20). **(C)** Immunohistochemical staining for CD68 shows that the histiocytes are positive (hematoxylin-eosinstain, original magnification × 20). **(D)** Under the 40x objective lens, the acid-fast special staining reveals red-stained bacilli (*Mycobacterium leprae*) within the histiocytes (hematoxylin-eosinstain, original magnification ×40).

Electrocardiogram, routine blood and urine tests, liver function, kidney function, electrolytes, and coagulation function were all normal. (HIV) test, syphilis serological reaction test (RPR), and hepatitis B surface antigen test were all negative. Bacterial Index (BI) test results: left eyebrow arch, 2+; right eyebrow arch, 2+; left earlobe, 3+; right earlobe, 3+; skin lesion, 4+. Histopathological examination of skin lesions on the left trunk: Epidermis normal, superficial dermis shows soft edema, dermal appendages are reduced, and there are clusters of tissue cells, foam cells, and lymphocytes infiltrating the blood vessels and appendages. Antiacid staining of the lesion on the left trunk showed positive acid-fast bacilli (Figures 2A–D).

### Differential diagnosis

1. Renal or cardiac causes of edema: This patient showed no clinical manifestations of renal or cardiac edema. Auxiliary examinations, such as urine routine, renal function, electrocardiogram, abdominal B-ultrasound, and cardiac ultrasound, are all normal, and thus can be ruled out.
2. Systemic lupus erythematosus (SLE) is often accompanied by photosensitivity, oral ulcers, hair loss, anemia, fever, and proteinuria. The main pathological changes in the skin tissue are interface dermatitis changes, which can be ruled out.
3. Syphilis (second stage syphilis): Positive syphilis serological tests (RPR and TPPA) are the basis for diagnosis. In this case, the RPR test result is negative; therefore, it can be ruled out.
4. Psoriasis: The scales of psoriasis are silver-white, and film phenomenon and punctate bleeding can be observed after scraping. The boundaries of the skin lesions are clear, and there is usually no obvious edema or numbness. Pathological examination showed incomplete keratinization of the stratum corneum, thinning of the granular layer, dilated capillary plexuses in the dermal papilla, and no acid-fast bacilli. The characteristics of the skin lesions and bacterial examination in this case do not support this.

**Figure 3:**
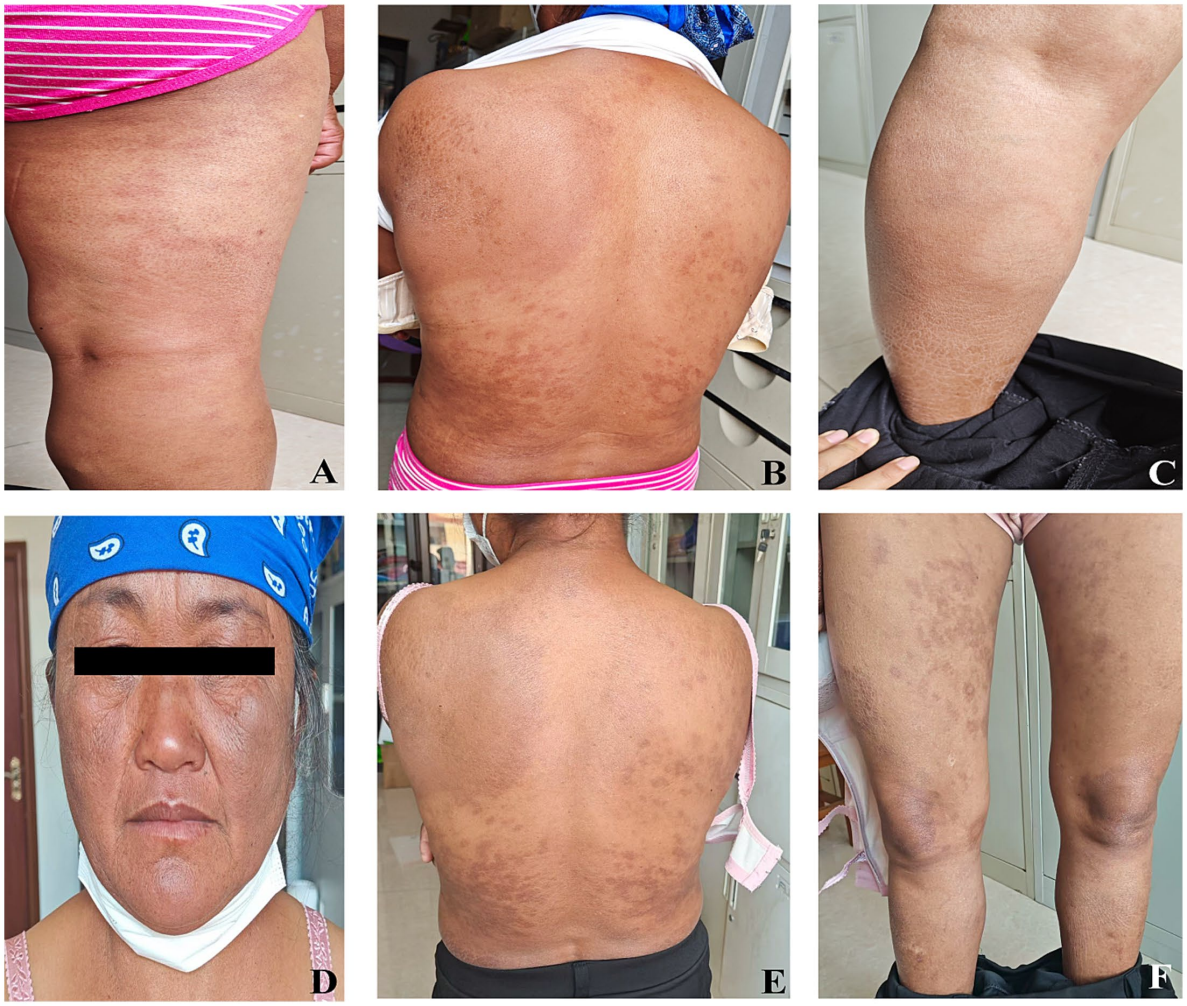
Skin manifestations in patients with marginal leprosy during follow-up after treatment. **(A–C)** Follow-up 1 year after treatment; **(D–F)** Follow-up 21 months after treatment.

Based on the above examination results and the fact that the patient has been living in a region with a high incidence of leprosy for a long time, the final diagnosis was marginal lepromatous granulomatous leprosy. Subsequently, the patient was transferred to the local disease prevention and control center in Pu’er City for treatment. The patient received standardized multi-bacillary combined chemotherapy (MDT) at the local disease prevention and control center to treat the patient. The local disease prevention and control center established an archive for the patient and conducted monthly follow-up records. Regular reexaminations of leprosy bacilli were conducted for the patient. At the end of the 1-year treatment, that is, in June 2024, the patient’s skin smear was examined for bacteria, with an average BI of 2.0. During the microscopic examination, a small number of intact leprosy bacilli and broken bacilli could be seen. At this time, the inflammation of the patient’s skin lesions decreased, and some areas showed a darker color of the skin lesions. Specific skin lesions are shown in Figures 3A–C. The patient continued to receive combined anti-leprosy chemotherapy. At 21 months of treatment, the patient’s skin smear was reexamined for bacteria again. The test was negative, and the clinical judgment was cured. At this time, the patient’s skin lesions still had obvious pigmentation, and the specific skin lesions are shown in Figures 3D–F.

## Discussion

Leprosy is a chronic infectious disease caused by *Mycobacterium leprae* that parasitizes the cytoplasm of macrophages and Schwann cells. Its main pathological features include granuloma formation and neurotropism. It mainly affects the skin and the peripheral nerves. Primary skin lesions usually present as erythema or hypopigmented macules with sensory loss (4). Several leprosy classification methods have been proposed. Rabello was the first to propose a classification system for leprosy, defining the undefined type of leprosy as the early form and introducing the concept of disease polarity - untreated cases can progress to tuberculous or tumor-like types. In 1953, this classification was further expanded, incorporating the less stable ‘boundary type’ subcategory (5, 6). Based on this classification, Ridley-Jopling further refined it, considering the correlations between skin lesion types, bacterial quantities, and cellular immune levels. They expanded the polarity system into five levels, namely leprosy can be classified as tuberculoid leprosy, borderline tuberculoid leprosy with intermediate borderline features, borderline papular leprosy, and papular leprosy. This has become a modern recognized classic classification system (7, 8). In addition, the WHO has developed a simple classification system for field use. Based on the number of skin sores, leprosy is divided into paucibacillary (PB, five or fewer skin sores) and multibacillary (MB, more than five skin sores) (9, 10). Based on this classification method, the patient was diagnosed with multi-bacillary borderline lepromatous leprosy.

Leprosy presents with complex and diverse clinical manifestations and has historically been referred to as the “Great Imitator,” making it difficult to diagnose, especially when patients present with atypical symptoms.

First, When the clinical manifestations of the skin lesions are diverse, such as erythema, papules, nodules, or maculopapular lesions without numbness or sensory abnormalities, misdiagnosis is common. Literature has shown that leprosy may be mistaken for mycosis fungoides, hemophagocy lymphohistiocytosis, sarcoidosis, or systemic lupus erythematosus (11–14). For example, a 55-year-old male presented with copper-colored papular lesions (0.3–0.5 cm in diameter) on the soles and red papules on the lower and upper legs, but no other symptoms, a presentation resembling secondary syphilis, but later considered leprosy (15). Another rare case involved a 36-year-old pregnant woman at 29 weeks of gestation, who developed pruritic papules over her body and large nodular lesions on her lower limbs without itching, initially misdiagnosed as pruritic urticarial papules and plaques of pregnancy (PUPPP) (16).

Second, when skin lesions present as hypopigmented or mildly discolored patches without numbness or inflammation, they may be misdiagnosed as vitiligo, tinea versicolor, or other dermatoses. In one case, a Nigerian immigrant to Italy developed widespread, pruritic, and poorly demarcated hypopigmented patches that tended to coalesce. After immunological, hematological, and allergological tests, biopsy, and empirical antifungal treatment, no definitive diagnosis was made. Only when numbness and sensory loss developed in the legs, and another biopsy with microbiological tests was performed, was the diagnosis of borderline lepromatous multibacillary leprosy confirmed (17)^.^

Third, patients may present without skin lesions, showing only isolated peripheral nerve involvement such as nerve thickening, numbness, or muscle weakness. When only one nerve is affected, it is easily misdiagnosed as peripheral neuritis, nerve compression syndrome, local trauma, or entrapment neuropathy. A neurologist in Italy reported a patient whose primary presentation was inflammatory myopathy, ultimately diagnosed with leprosy (18). A 38-year-old man presented with radiating pain in the right leg and a tender, swollen area below the fibular head with radiation to the lateral leg, no skin lesions, and mild sensory deficit, eventually diagnosed with leprosy (19). In another rare case described by Maurya et al., the patient had only sensory symptoms, such as numbness over the dorsum of the right foot, without detectable muscle weakness or skin lesions. Deep tendon reflexes were normal (20). Studies have shown that 45–60% of leprosy cases involve the muscles, often without obvious symptoms. Pathological changes may be neurogenic or myogenic in nature. Approximately 30% of cases present with myositis, yet only 25% show detectable *M. leprae* in muscle tissue (21–23).

Fourth, when patients present with other atypical symptoms such as joint pain or synovitis, they may be misdiagnosed with rheumatoid arthritis. Ocular manifestations, such as iritis or keratitis, can be the initial symptoms leading to misdiagnosis. Lymphadenopathy may be mistaken for tuberculous lymphadenitis or lymphomas. In some cases, multisystem involvement occurs, with fever, weight loss, anemia, and skin rashes, making the diagnosis more difficult. One case involved a 43-year-old woman with recurrent fever, superficial lymphadenopathy, and multiple facial and body rashes, without pain, itching, or burning. She also had sensory deficits in the eyes, hands, and feet, although there was no motor dysfunction. The patient was eventually diagnosed (24). Another patient presented with unexplained fever, anemia, wasting syndrome, and neuropathy and was ultimately diagnosed with leprosy (25). These cases suggest that bone marrow involvement should be considered when cytopenia and a positive epidemiological history are observed.

The immune response of the borderline type of leprosy with granulomas lies between the tuberculous type and the tumor type. Previous reported cases have shown the diversity of their clinical manifestations, particularly in terms of edema-related and other atypical symptoms (see Table 1 and Supplementary Table S1 for details) (26–45). The clinical manifestations are somewhat unstable and can transform into either the tuberculous type or the tumor type. In the reported cases of this study, the patients presented with generalized edema as the initial symptom, followed by skin lesions and numbness in the hands and feet as the disease progressed. There have been cases with generalized edema reported in the past, but they were not the initial symptoms. For instance, the case reported by Sujoy Ghosh et al. (32) occurred during the course of the disease. There are also reports of cases where edema appeared in the hands, feet, and limbs as the initial symptom, such as those by Barman KD, Moulick A, and Guerra MG (37, 42, 44). However, no studies have reported cases with generalized edema as the initial symptom. Some of the above reports and this case indicate that edema is one of the clinical symptoms that occur in leprosy patients, especially in the form of swelling in the hands and feet, and generalized edema can also occur in leprosy patients.

**Table 1:** Atypical clinical manifestations involving edema in previously reported cases of borderline lepromatous leprosy.

Author	Age (yrs)	Year	Initial symptoms	Other accompanying symptoms
Li Lu et al. (this case)	52	2025	Generalized edema involving the face, trunk, and extremities, accompanied by mild numbness in the hands and feet.	Erythema emerging on the lumbar region (with mild pruritus) and progressively spreading to the extremities, buttocks, abdomen, back, neck, and face; aggravated numbness in the hands and feet; scattered, variably sized hypopigmented macules; a large infiltrated plaque in the lumbar area; mild facial and earlobe swelling; pitting edema on the dorsal surfaces of the hands, lower legs, and ankles; dry, scaly skin lesions with blurred borders and slightly indurated lesional areas.
Ghosh et al. (32)	27	2010	The sensation in both forearms has weakened.	The condition has progressed, with generalized swelling (not the initial symptoms); red scaly patches on both legs and forearms; swelling of the palate, with two papular nodular lesions on the hard palate, approximately 1 cm in size, without pain, no bleeding, and no purulent discharge.
Fernando et al (37)	48	2024	Edema of both lower extremities.	"Fish-scale-like" rashes on both hands and feet.
Moulick et al (42)	23	2013	Non-pitting edema of the limbs	Associated with arthritis of the wrist, elbow, ankle and knee; accompanied by diffuse scaly skin lesions (ichthyosis)
Guerra et al (44)	68	2019	Joint pain and swelling in the limbs	With swelling and pain in both bilateral MCPJ, PIPJ, tarsometatarsal joints and metatarsophalangeal joints; localized edema in both hands and feet; gradually developing loss of sensitivity in the form of glove and sock patterns; and red patches/purple patches with papules and plaques in the trunk and limbs.

MCPJ, metacarpophalangeal joints; PIPJ, proximal interphalangeal joints.

This may result from early immune responses involving a Th17/Treg imbalance, which leads to the release of inflammatory mediators. These mediators cause local vasodilation and increased vascular permeability, resulting in fluid leakage into interstitial tissues and subsequent swelling. Studies suggest that leprosy patients have reduced suppressive activity of regulatory T cells (Tregs) and increased activity of T helper 17 (Th17) cells. Th17 cells secrete cytokines like IL-17, IL-21, and IL-22, which promote neutrophil infiltration and inflammatory swelling (46–48). In addition, immune complex deposition in vessels contributes to further inflammation and secondary vasculitis, damaging vessel walls, impeding venous return, and preventing metabolic waste removal, thus worsening edema (49–51). These mechanisms may explain the facial, trunk, and limb edema observed in the present case, which may be misdiagnosed as cardiac or renal causes of edema.

Beyond edema, borderline lepromatous leprosy can present with various other atypical symptoms, such as skin ulcers, nerve thickening, ocular involvement, testicular pain, nasal congestion, and nipple abnormalities, which are summarized in Supplementary Table S1 for comprehensive reference.

## Patient insights and recommendations

Although leprosy is curable and preventable, its historical stigma persists, fueling public misunderstandings and discrimination. This psychological burden often delays or deters patients from seeking care. To mitigate these challenges, a multi-faceted strategy is essential.

### Patient-centered support

Designated leprosy treatment centers integrate disease counseling and psychological interventions into routine care, helping patients cope with stigma. Additionally, fostering patient support networks (e.g., peer groups) empowers individuals to share experiences and rebuild confidence.

### Community and policy interventions

Public education campaigns—leveraging media, online platforms, and intensified outreach during events like World Leprosy Day—disseminate key messages: leprosy is treatable, non-feared, and preventable. Concurrently, strengthened policies (medical assistance, living subsidies, and legal protection of patients’ rights) alleviate economic burdens and combat social exclusion, facilitating patients’ reintegration.

For clinicians: In endemic regions, unexplained edema/erythema warrants proactive assessment of sensory deficits, coupled with skin biopsy and acid-fast staining. For health authorities: Establish joint screening mechanisms for leprosy and mimicking conditions (e.g., renal disease, rheumatism) to improve early detection and reduce misdiagnosis.

## Conclusion

These cases demonstrate the diversity and complexity of the clinical manifestations of leprosy. Although the prevalence of leprosy worldwide is currently low, with ease of international travel, it is still possible for leprosy cases to occur anywhere. This requires clinicians to enhance their understanding of the various presentations of leprosy and to be well prepared for diagnosis.

## Data Availability

The original contributions presented in the study are included in the article/Supplementary material, further inquiries can be directed to the corresponding author.

## References

[1] Anderson BE. The netter collection of medical illustrations. Beijing: Science Press (2017).

[2] Panigrahi R Priyadarshini SR Sahoo PK Alam T Saeed S Hasan S. Lepromatous leprosy manifesting as chronic macrocheilia: report of a rare case. Cureus. (2023) 15:e47859. doi: 10.7759/cureus.47859 38021977 PMC10680308

[3] Sun PW Wang L Wang HS Yan LB Yu MW. Epidemiological characteristics of leprosy in China from 2016 to 2020. Chin J Dermatol. (2023) 56:204–9. doi: 10.35541/cjd.20220512

[4] Bolognia JL Jorizzo JL Rapini RP. Dermatology. Beijing: Peking University Medical Press (2010).

[5] World Health Organization. (2022). Leprosy. Available online at: https://www.who.int/en/news-room/fact-sheets/detail/leprosy (Accessed August 02, 2025).

[6] Oliveira MBB Diniz LM. Leprosy among children under 15 years of age: literature review. An Bras Dermatol. (2016) 91:196–203. doi: 10.1590/abd1806-4841.20163661, PMID: 27192519 PMC4861567

[7] Ridley DS Jopling WH. Classification of leprosy according to immunity. A five-group system. Int J Lepr Other Mycobact Dis. (1966) 34:255–73. 5950347

[8] World Health Organization. (2022) Guidelines for the diagnosis, treatment and prevention of leprosy 2022. Available online at: https://apps.who.int/iris/bitstream/handle/10665/274127/9789290226383-eng.pdf (Accessed August 02, 2025).

[9] World Health Organization. (2018). Guidelines for the diagnosis, treatment and prevention of leprosy. Available online at: https://apps.who.int/iris/handle/10665/274127 (Accessed August 03, 2025).

[10] WHO. WHO Expert Committee on Leprosy. World Health Organ Tech Rep Ser. (1998) 874:1–43. 9627517

[11] Liu J Wen Y Xing Y Wang S. Borderline tuberculoid leprosy mimicking sarcoidosis: a case report. Medicine. (2018) 97:e11616. doi: 10.1097/MD.0000000000011616, PMID: 30095620 PMC6133579

[12] Rodríguez-Acosta ED Esquivel-Pedraza L Saeb-Lima M Arenas-Guzmán R Granados-Arriola J Domínguez-Cherit J. Borderline tuberculoid leprosy mimicking mycosis fungoides. Skinmed. (2013) 11:379–81. 24517048

[13] Zeng XZ Wang YN Wang JS Wu L Zhang J Wei Q. A case of lepromatous leprosy complicated by hemophagocytosis misdiagnosed ashemophagocytic lymphohistiocytos is. Int J Infect Dis. (2014) 23:28–30. doi: 10.1016/j.ijid.2014.02.004, PMID: 24657272

[14] Zhao Y Huang S Wu T. *Mycobacterium leprae* infection with rash misdiagnosed as a flare-up of systemic lupus erythematosus: a case report. Diagn Microbiol Infect Dis. (2025) 111:116622. doi: 10.1016/j.diagmicrobio.2024.116622. Epub 2024 Nov 27., PMID: 39637678

[15] Chaabane H Ayedi L Bahloul E Amouri M Masmoudi A Boudawara T. Une lèpre lépromateuse révélée par des papules palmo-plantaires Lepromatous leprosy revealed by palmoplantar papular lesions. Ann Dermatol Venereol. (2015) 142:616–8. doi: 10.1016/j.annder.2015.04.013 26024863

[16] Sánchez-Orta A Albízuri Prado MF González Pessolani T Sendagorta CE. Pruritic lesions during pregnancy: an unusual presentation of a rare variant of multibacillary leprosy. Actas Dermosifiliogr. (2016) 107:352–4. doi: 10.1016/j.ad.2015.08.010 26739118

[17] Asperges E Bagnarino J Ancarani C Baggini G Filardo M Monzillo V. A case of leprosy in a nonendemic country. Int J Infect Dis. (2024) 143:107004. doi: 10.1016/j.ijid.2024.107004, PMID: 38479578

[18] Liguori R Terlizzi R Giannoccaro MP Amati A Foschini MP Parodi A. An inflammatory myopathy unmasks a case of leprosy in an Italian patient. J Neurol. (2015) 262:2179–81. doi: 10.1007/s00415-015-7864-7, PMID: 26233690

[19] Jain S Ramesh V Antil N. Painful swelling on the side of the knee. Clin Exp Dermatol. (2015) 40:586–8. doi: 10.1111/ced.12607, PMID: 25678069

[20] Maurya PK Kulshreshtha D Singh AK Thacker AK Malhotra KP. Isolated superficial peroneal neuropathy: a rare presentation of Hansen's disease(leprosy). QJM. (2015) 108:419–20. doi: 10.1093/qjmed/hcu131, PMID: 24939189

[21] Werneck LC Teive HA Scola RH. Muscle involvement in leprosy. Study of the anterior tibial muscle in 40 patients. Arq Neuropsiquiatr. (1999) 57:723–34. doi: 10.1590/s0004-282x1999000500001, PMID: 10751905

[22] Gupta JC Jesupadam T Gupta MC Gupta DK. A histopathologic study of striated muscle biopsies in leprosy. Int J Lepr Other Mycobact Dis. (1975) 43:348–55. 776842

[23] Salvi S Chopra A. Leprosy in a rheumatology setting: a challenging mimic to expose. Clin Rheumatol. (2013) 32:1557–63. doi: 10.1007/s10067-013-2276-5, PMID: 23645094 PMC3778233

[24] Zhang Y Lei X Lu J. Next-generation sequencing-assisted diagnosis of a case of leprosy misdiagnosed as erythema multiforme. Ann Clin Microbiol Antimicrob. (2022) 21:40. doi: 10.1186/s12941-022-00532-4, PMID: 36071525 PMC9454213

[25] Santana MAO da Costa WVT Celestino IC Dos Santos DF Dornelas BC Pavelka MM. Fever of unknown origin, wasting syndrome and bone marrow involvement: a leprosy case report. Front Immunol. (2022) 13:916319. doi: 10.3389/fimmu.2022.916319, PMID: 35874693 PMC9300819

[26] Hutahaean GD Susanto M Napitupulu T Pangihutan-Siahaan AM Wirjomartani BA. Childhood borderline lepromatous leprosy: a case report. Turk J Pediatr. (2023) 65:862–7. doi: 10.24953/turkjped.2023.138, PMID: 37853977

[27] Chiriboga G Guo Q Zuberi E Powers HR Rueda Prada L. Erythema nodosum leprosum in a patient with borderline lepromatous leprosy: a case report. Infect Dis Rep. (2025) 17:83. doi: 10.3390/idr17040083, PMID: 40700329 PMC12285923

[28] Reis VHMD Miola AC Ferreira CAZ Milagres SP Lastória JC Schmitt JV. Borderline lepromatous leprosy with lymph node infiltration: dermatology helps to clarify challenging diagnoses. An Bras Dermatol. (2025) 100:615–8. doi: 10.1016/j.abd.2024.10.004 40189967 PMC12234200

[29] Gunawan H Utami F Achdiat PA Avriyanti E Hindritiani R Suwarsa O. A unique case of borderline lepromatous leprosy with psoriasis-like lesions all over the body and mycosis fungoides-like lesions on the face. J Clin Tuberc Other Mycobact Dis. (2019) 17:100134. doi: 10.1016/j.jctube.2019.100134, PMID: 31867445 PMC6904841

[30] Anand P Bharadwaj S Ganguly S Rao S. Palatal perforation in a patient with borderline lepromatous leprosy: leprosy still not eradicated. BMJ Case Rep. (2022) 15:e251798. doi: 10.1136/bcr-2022-251798, PMID: 36192029 PMC9535138

[31] Dey B Gochhait D Prabhakaran N Chandrashekar L Behera B. A rare case of coexistence of borderline lepromatous leprosy with tuberculosis Verrucosa Cutis. Case Rep Infect Dis. (2016) 2016:6896. doi: 10.1155/2016/1746896, PMID: 28003920 PMC5143736

[32] Ghosh S Gadda RB Vengal M Pai KM Balachandran C Rao R. Oro-facial aspects of leprosy: report of two cases with literature review. Med Oral Patol Oral Cir Bucal. (2010) 15:e459–62. doi: 10.4317/medoral.15.e459, PMID: 20038902

[33] Bezerra NTC Schettini APM Leturiondo AL Mathias LHMT. Case for diagnosis. Erythematous and infiltrated plaques in the infrahyoid region. An Bras Dermatol. (2021) 96:97–9. doi: 10.1016/j.abd.2020.03.022, PMID: 33288364 PMC7838112

[34] Sena CO Sr Goulart IMB Justino Sena PC Justino Omar JC Dornelas BC. Leprosy mimicking thrombangiitis obliterans (Buerger's disease): a case study. Cureus. (2025) 17:e80822. doi: 10.7759/cureus.80822, PMID: 40255772 PMC12007437

[35] Yang S Makredes M O'Donnell P Levin NA. A case of Hansen disease presenting as tinea versicolor. Dermatol Online J. (2013) 19:7. doi: 10.5070/D365h88318 24021367

[36] Prabha N Mahajan VK Sharma SK Sharma V Chauhan PS Mehta KS. Optic nerve involvement in a borderline lepromatous leprosy patient on multidrug therapy. Lepr Rev. (2013) 84:316–21. doi: 10.47276/lr.84.4.316, PMID: 24745131

[37] Fernando N Welhenge C Premaratna R Uwyse A. Borderline lepromatous leprosy: a case report. Asian Pac J Trop Med. (2024) 17:329–32. doi: 10.4103/apjtm.apjtm_874_23

[38] Pandhi D Verma P Sharma S Dhawan AK. Borderline-lepromatous leprosy manifesting as granulomatous mastitis. Lepr Rev. (2012) 83:202–4. doi: 10.47276/lr.83.2.202, PMID: 22997696

[39] Barman KD Gupta U Saify K Luthra S. Sub-polar lepromatous leprosy presenting as urticarial wheals: a case report. Indian J Lepr. (2004) 76:223–8. 15835607

[40] Abraham S Ebenezer GJ Jesudasan K. Diffuse alopecia of the scalp in borderline-lepromatous leprosy in an Indian patient. Lepr Rev. (1997) 68:336–40. doi: 10.5935/0305-7518.19970043, PMID: 9503871

[41] Acharya PV Gupta OP Nigam SP Agrawal RV. Reactive episodes in borderline lepromatous leprosy--a case report. Lepr India. (1976) 48:311–3. 1022972

[42] Moulick A Jana A Sarkar N Guha P Mahapatra C Lallawmzuala K. Non pitting edema, arthritis and ichthyosis; presenting manifestation of leprosy. Indian J Lepr. (2013) 85:83–6. 24236367

[43] Sumangala S Nikfekr E George J Holmes CW. Leprosy neuropathy masquerading as cellulitis. Postgrad Med J. (2019) 95:225–6. doi: 10.1136/postgradmedj-2019-136392, PMID: 30918118

[44] Guerra MG Videira TMFC Morais HAG Santos TCRN Taipa RJF Abreu MA. Leprosy presenting as remitting seronegative symmetrical synovitis with pitting oedema syndrome-a case report. BMC Infect Dis. (2019) 19:455. doi: 10.1186/s12879-019-4098-9, PMID: 31117984 PMC6530132

[45] Qu H Tu P Yu J. Swollen hand joints with asymptomatic nodules on the skin. JAMA Dermatol. (2017) 153:313–4. doi: 10.1001/jamadermatol.2016.4261, PMID: 27926765

[46] Aride DB Dalmaso BF Moulin ACS Moulaz IR Machado KLLL. Leprosy mimicking autoimmune diseases: a case series. Clin Exp Rheumatol. (2024) 42:746–51. doi: 10.55563/clinexprheumatol/ov3kgl, PMID: 38372720

[47] Silva MJA Silva CS Brasil TP Alves AK Dos Santos EC Frota CC. An update on leprosy immunopathogenesis: systematic review. Front Immunol. (2024) 15:1416177. doi: 10.3389/fimmu.2024.1416177, PMID: 39308868 PMC11412872

[48] Grijsen ML Nguyen TH Pinheiro RO Singh P Lambert SM Walker SL. Leprosy. Nat Rev Dis Primers. (2024) 10:90. doi: 10.1038/s41572-024-00575-1, PMID: 39609422

[49] Thomas M Pratibha J Emmanuel M Neivitro AN. Recurrent ulcers: a diagnostic challenge. Indian J Dermatol. (2013) 58:329. doi: 10.4103/0019-5154.113979, PMID: 23919035 PMC3726912

[50] Sheetal S Arvind C. Lest we forget Hansen's disease (leprosy): an unusual presentation with an acute onset of inflammatory polyarthritis and the rheumatology experience. Int J Rheum Dis. (2009) 12:64–9. doi: 10.1111/j.1756-185X.2009.01382.x, PMID: 20374320

[51] Gupta S Li C Thallapally VK Sharma P Nahas J. Chronic hand swelling and Dactylitis in leprosy: a case report and review of the literature. Cureus. (2021) 13:e13451. doi: 10.7759/cureus.13451, PMID: 33767935 PMC7983738

