# Supplementary material for: Case Report: A case of borderline lepromatous leprosy and literature review—key clues to expand clinical diagnostic thinking
Source: Front Med (Lausanne). 2025 Sep 16;12:1636112. doi: 10.3389/fmed.2025.1636112 (PMC12481899; doi:10.3389/fmed.2025.1636112)
Supplement: Supplementary file 1 [file Supplementary_file_1.doc]

**Table S1. Other non-typical clinical manifestations of borderline lepromatous leprosy reported in previous literature**

| **Author** | **Age**  **(yrs)** | **year** | **Initial symptoms** | **Other accompanying symptoms** | **General symptoms** | **Reference Number** |
| --- | --- | --- | --- | --- | --- | --- |
| Hutahaen GD et al. | 9 | 2023 | The three fingers on the left hand are stiff. | Discolored patches on the back and buttocks, without pain or itching. | Not mentioned | 26 |
| Guido Chiriboga et al. | 62 | 2025 | Upper limb erythema and patches gradually spread to the face, chest, abdomen and lower limbs. | Swelling and numbness in the hands and feet; persistent cold sensation in the nasal cavity. During the treatment, there were repeated cases of facial cellulitis and systemic erythema nodosum. | Not mentioned | 27 |
| Victor Henrique Murback Dos Rei et al. | 48 | 2025 | Testicular pain radiates to the groin area. | Enlargement of bilateral inguinal lymph nodes. | accompanied by chills, insomnia, night sweats, weight loss. | 28 |
| Hendra Gunawan et al | 43 | 2019 | There is a pigment-depleted circular rash with no subjective symptoms on the upper left back area. | Facial patches and lumps; generalized scaly erythema, papules, and plaques; edema of the upper and lower extremities, decreased sensation in the right lower extremity; enlargement of the right ulnar nerve and bilateral common peroneal nerves, without tenderness. | Not mentioned | 29 |
| Preetha Anand et al | 30 | 2022 | Nasal congestion, intermittent nosebleeds, and tingling sensations in the hands and feet. | Hard palate perforation (a 7mm diameter orifice at the anterior 1/3 of the hard palate, with no pain and no purulent discharge). Pigment loss patches were observed on the trunk and limbs. | Not mentioned | 30 |
| Dey B.et al. | 29 | 2016 | There are asymptomatic skin-colored raised lesions on both earlobes. | Multiple soft, juicy, painless, skin-colored superficial nodules appeared on the face, earlobes, forearms, back and chest; multiple verrucous plaques were found on the right ankle; the large nerves of both ears and the right common peroneal nerve were thickened, and the left posterior tibial nerve was tender. | accompanied by a general feeling of intense heat and a low fever. | 31 |
| Natália Tenório Cavalcante Bezerra.et al | 46 | 2021 | Two red infiltrative plaques appeared in the area below the hyoid bone. | The skin lesions are accompanied by changes in temperature sensation, tactile sensation and sensitivity to pain. | Diarrhea, weight loss. | 33 |
| [Caio O Sena Sr](https://pubmed.ncbi.nlm.nih.gov/?term=).et al | 88 | 2025 | Lower extremity skin ulcer. | Multiple black skin ulcers in both lower extremities; linear skin scars can be seen on the legs, and dry gangrene has occurred in the distal phalanges of the right toes II and III, and the left toes I, II, and III. | Not mentioned | 34 |
| Yang S.et al | 24 | 2013 | Symptom-free hypopigmented patches and plaques on the upper body. | Gradually, numbness in the left arm and loss of pain sensation occurred. | Not mentioned | 35 |
| Prabha.et al. | 47 | 2013 | There is a single lesion in the left orbital region that appears to be a typical Borderline Border (BB) type leprosy lesion. | Painless vision loss occurred successively in both the left and right eyes. | Not mentioned | 36 |
| Pandhi D.et al | 35 | 2012 | The left nipple shows no symptoms of redness or thickening, but there is breast development. | Red spots, papules and patches can be seen on the back, chest and limbs. | Not mentioned | 38 |
| Barman KD.et al | 40 | 2004 | Scattered mild red spots all over the body. | Symmetrical distribution of urticarial wheals. Thickening of the right ulnar nerve and common peroneal nerve.。 | Not mentioned | 39 |
| Abraham S.et al | 64 | 1997 | The initial symptoms were not explicitly mentioned. | The patient did not receive regular treatment and the disease persisted for over 20 years. At the last visit, the patient presented with diffuse infiltration, dryness, and scales on the trunk and limbs; depigmented scars; loss of bilateral eyebrows, extensive hair loss; decreased sensation in the limbs, left foot ulcer, left foot drooping, and "rabbit eye" on the right side; and swelling of the lymph nodes under the left occipital region. The diagnosis changed from BL to LL. | Not mentioned | 40 |
| Acharya PV.et al | 37 | 1976 | The entire body has changed into a silvery white scaly appearance. | Some areas have scars, and there is an oily crusty reaction on the cheeks. After treatment, a pustular reaction occurred. | Not mentioned | 41 |
| Sumangala S.et al | 41 | 2019 | The left forearm is swollen, accompanied by severe local pain and redness. | The left hand has a claw-like shape; there are maculopapular rashes on the right arm and the trunk. | Not mentioned | 43 |
| Qu H.et al | 40 | 2016 | Eyelashes, eyebrows, beard and hair gradually fall out. | Hand joint swelling and pain; asymptomatic nodules on the upper arm; osteoarthritis, synovitis, flexor tendon sheath inflammation, bone erosion; enlarged axillary lymph nodes, cervical lymph nodes, inguinal lymph nodes, enlarged spleen; uveitis | Not mentioned | 45 |
